# Kinetic Study of DNA Modification by Phthalocyanine Derivative of the Oligonucleotide

**DOI:** 10.1155/BCA/2006/23560

**Published:** 2006-12-18

**Authors:** Alexandra A. Kuznetsova, Alexander A. Chernonosov, Nikita A. Kuznetsov, Vladimir V. Koval, Dmitri G. Knorre, Olga S. Fedorova

**Affiliations:** ^1^Institute of Chemical Biology and Fundamental Medicine, Siberian Branch of the Russian Academy of Sciences, Novosibirsk 630090, Russia; ^2^Department of Natural Sciences, Novosibirsk State University, Novosibirsk 630090, Russia; ^3^Institute of Human Ecology, Siberian Branch of the Russian Academy of Sciences, Kemerovo 650099, Russia

## Abstract

Design of chemically modified oligonucleotides for regulation of gene expression has attracted considerable attention over the last decades. One actively pursued approach involves antisense or antigene constructs carrying reactive groups, many of these based on transition metal complexes. The complexes of Co(II) with phthalocyanines are extremely good catalysts of oxidation of organic compounds with molecular oxygen and hydrogen peroxide. In this study, we have investigated the kinetics and thermodynamics of sequence-specific modification of DNA with deoxyribooligonucleotide linked to Co(II)-tetracarboxyphthalocyanine (PtcCo(II)) in the presence of H_2_O_2_.

## INTRODUCTION

The principles underlining antisense and antigene strategies are conceptually very simple and straightforward. The use of a complementary sequence can inhibit the expression of a specific mRNA, breaking the transfer of genetic information from DNA to protein. The development of oligonucleotide derivatives that can bind sequence specifically to unique sites in mRNA or genomic DNA and modify the target to a great extent or even completely may have major implications for the treatment of hereditary diseases, cancers, and viral infections [1–4]. This approach, relying on sequence-specific targeting of reactive compounds, was initially called “complementary addressed modification of nucleic acids” [5]. Kinetic studies of these processes provide quantitative estimates of the selectivity and efficiency of the modification of nucleic acids.

Oligonucleotides derivatives carrying catalytic groups can achieve multiple turnover and convert many target molecules. Complexes of porphyrins and phthalocyanines with the transition metal ions (Fe, Co, etc.) are considered among the most efficient catalytic groups for this purpose [6].

**Scheme 1:**
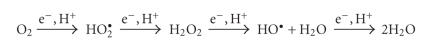
The reduction of O_2_ molecule.

Molecular oxygen is a four-electron oxidant; its reduction to H_2_O is presented in Scheme 1. As one-step two- or four-electron reduction of O_2_ is forbidden by spin exclusion principle, direct oxidation of organic substrates with a triplet oxygen molecule does not occur. This difficulty can be overcome either by converting the oxygen molecule from its ground triplet state to the excited singlet state or by sequential one-electron reduction to H_2_O catalyzed by transition metal ions. Among reactive oxygen species formed in the latter pathway (Scheme 1), hydroxyl radical ^•^OH is the strongest oxidant [7, 8] capable of damaging various cell constituents including DNA [9].

It was recently shown that complexes of phthalocyanines with Co(II) and Fe(II) are very efficient catalysts of oxidation of various organic substrates with molecular oxygen and hydrogen peroxide [6]. If molecular oxygen is the oxidant, a reducing agent is also required to convert the metal ion to a low valent state.

**Figure 1:**
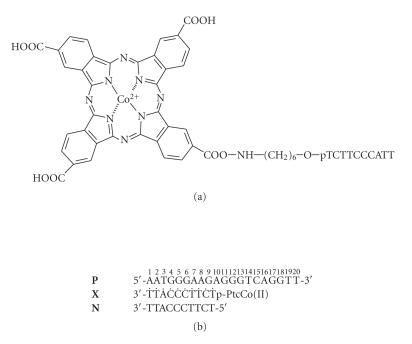
(a) Structure of the phthalocyanine conjugate; (b) the sequence of oligonucleotide duplex used for the complementary-addressed DNA modification.

The main goal of this paper was to determine the kinetic features of the interaction of the PtcCo(II)-oligonucleotide conjugate with single-stranded DNA. Earlier we have shown that the system of O_2_, a PtcCo(II) conjugate, and a reductant can oxidize DNA [10], but this reaction is very slow. Therefore, in the present study H_2_O_2_ was taken as an oxidant instead of molecular oxygen. In this case, the first unfavorable step in Scheme 1 is left out and the target oxidation is accelerated significantly, allowing one to estimate the contribution of other stages to the oxidation process. In addition to being the source of ^•^OH radicals, H_2_O_2_ serves as the reducer of the oxidized form of catalyst. The structures of the metallophthalocyanine conjugate and the target DNA are presented in Figure 1.

To gain a deeper insight into the reactivity of PtcCo(II)-group and the mechanism of the DNA target modification by the PtcCo(II)-oligonucleotide conjugate, we have studied separately the different stages of this process. The first step was duplex formation between the target and the conjugate (**X**) or a nonmodified oligonucleotide (**N**). This equilibrium was studied by stopped-flow kinetics and UV melting curve analysis. Degradation of the PtcCo(II) residue in the conjugate in the presence of H_2_O_2_ was detected by changes in the absorption spectrum of this moiety during the reaction. The products of the catalytic oxidative modification of the target deoxyribooligonucleotide were registered by gel electrophoresis after treatment with piperidine (to reveal alkali-labile sites) or *Escherichia coli* Fpg protein (to reveal 8-oxoguanine and abasic sites).

## EXPERIMENTAL

### Chemicals and reagents

Acrylamide, N,N′-methylene-bisacrylamide, urea, acetonitrile, DMF (Fluka, Switzerland), Tris-HCl, and piperidine (Sigma-Aldrich, USA) were used. All solutions were prepared with double-distilled water using ultrapure reagents. Hydrogen peroxide (stabilized, > 30%) was purchased from Fluka. T4 polynucleotide kinase was purchased from Sibenzyme (Russia). Fpg protein from *E coli* was overexpressed, purified, assayed, and stored as described previously [11]. [*γ*-^32^P]ATP (> 3000 Ci/mmol) was purchased from Biosan (Russia). All binding and modification experiments were carried out at 25°C in a buffer containing 50 mM Tris-HCl (pH = 7.5), 0.1 M NaCl, 10 mM EDTA.

### Oligonucleotides and the conjugate

The 20 nt and 10 nt deoxyribonucleotides d(AATGGGAAGAGGGTCAGGTT), d(TCTTCCCATT), and pd(TCTTCCCATT) were synthesized on an ASM-700 automated synthesizer (Biosset, Russia) from phosphoramidites purchased from Glen Research (USA) according to the manufacturer's protocol. The oligonucleotides were deprotected with ammonium hydroxide and purified by ion exchange HPLC on a Nucleosil 100-10 N(CH_3_)_2_ column followed by reverse-phase HPLC on a Nucleosil 100-10 C_18_ column (both 4.6 × 250 mm, purchased from Macherey-Nagel, Germany). The purity of the oligonucleotides exceeded 98%, as estimated by electrophoresis in 20% denaturing polyacrylamide gel and staining with Stains-All dye (Sigma-Aldrich, USA). Concentrations of the oligonucleotides were determined from their absorbance at 260 nm [12].

The conjugate PtcCo(II)-NH-(CH_2_)_6_-O-pd(TCTTCCCATT) was synthesized using a previously reported solid-phase method [13] with 40% yield. The formation of the conjugate as the main reaction product was confirmed by MALDI-TOF. The mass spectrum contained the peak with m/z = 3839.52 corresponding to the mass of the molecular ion [M + H]^+^ (the calculated molecular mass of the conjugate is 3838.62 g/mol).

### Stopped-flow experiments

Stopped-flow measurements with UV absorbance detection were carried out using a model SX.18MV stopped-flow spectrometer (Applied Photophysics, UK) fitted with a 150 W Xe arc lamp and a 1 cm path length cell. The optical density of the solution was recorded at 255 nm. Solution of the target oligonucleotide **P** in one syringe was rapidly mixed with a solution of the conjugate **X** or nonmodified oligonucleotide **N** in another syringe. The concentration of **P**, **X**, and **N** were varied between 1.0 and 7.5 *μ*M. Concentrations of reactants reported are those in the reaction chamber after mixing. Typically, each trace shown is the average of four or more individual experiments. The dead time of the instrument was 1.4 ms.

### UV melting experiments

Absorbance versus temperature profiles were recorded at 260, 270, 280, and 300 nm using the optical detector of a Milikhrom chromatograph (Russia) connected to a PC. Melting profiles were obtained by heating at 0.5–0.9°C/min. The concentration of each strand was 5.0 × 10^–6^ M and the cell volume was 2 *μ*l. The data were analyzed taking into account the thermal expansion coefficient of water. The differential curves were obtained from the integral ones by calculating the increment of the optical density per 1°C of temperature growth. Thermodynamic parameters (ΔH^0^, ΔS^0^) were calculated according to [14].

### 5′-Terminal phosphorylation

The 5′-end of the oligonucleotide **P** was ^32^P-labeled using the standard procedure with T4-polynucleotide kinase and [*γ*-^32^P]ATP (> 3000 Ci/mmol) [15].

### Degradation of the phthalocyanine group attached to oligonucleotide

The change of the optical density of the solution at 682 nm where only the phthalocyanine group of the conjugate absorbs was followed using a Shimadzu UV2100 spectrophotometer. The concentrations of the conjugate and hydrogen peroxide were changed in the ranges 8.0 × 10^–6^ – 12.0 × 10^–6^ M and 5.0 × 10^–4^ – 1.0 × 10^–2^ M, respectively.

### Modification of the target oligonucleotide

Modification of the [^32^P]-labeled **P** was carried out in the presence of hydrogen peroxide. The concentration of **P** in the reaction mixture was 1.0 × 10^–8^ M, concentrations of the conjugate and hydrogen peroxide were changed in the range 0.4×10^–6^ –1.0×10^–5^ M and 1.0×10^–3^ –1.0×10^–1^ M, respectively. The reaction was initiated by adding H_2_O_2_. Aliquots were taken from the reaction mixture at different times and were immediately transferred into polypropylene tubes containing 200 *μ*l of 2% LiClO_4_ in acetone. The precipitate was pelleted by centrifugation, washed twice with 80% ethanol and once with acetone, and dried in vacuum. The samples were then treated with piperidine or Fpg. The products of the modification were separated by 20% PAGE in the presence of 7 M urea. After electrophoresis, the gel was exposed to CP-BU X-ray film (Agfa-Gevaert, Belgium) for 10–20 h at –10°C. The autoradiograms were scanned and quantified using Gel-Pro Analyzer v4.0 software (Media Cybernetics, MD). The extent of modification was calculated as the ratio of the integral intensity of the spot corresponding to the modification product to the sum of the intensities of the spots corresponding to the targets and the products.

### Piperidine treatment

The precipitates were dissolved in 100 *μ*l of 1 M piperidine (pH 12) and incubated for 30 min at 95°C [16]. After that the reaction mixtures were precipitated with 10 volumes of 2% LiClO_4_ in acetone, washed with 80% ethanol, then with acetone, dried under vacuum, and dissolved in 2–4 *μ*l of the gel-loading dye containing 0.1% bromophenol blue, 0.1% xylene cyanol FF, and 7 M urea.

### Fpg treatment

The precipitates were dissolved in 2 *μ*l of the buffer containing 50 mM Tris-HCl (pH 7, 5), 50 mM KCl, 1 mM EDTA, 1 mM dithiothreitol, 9% glycerol, and 9 × 10^–6^ M Fpg. After incubation of the reaction mixtures at 25°C for 2 h, 2 *μ*l of the gel-loading dye were added to each probe.

### Analysis of the kinetic curves

Kinetic parameters were obtained by numerical fitting using Origin v7.0 (OriginLab, USA), DynaFit (BioKin, USA) [17], Scientist, Simplex, and SigmaPlot v9.0 software.

## RESULTS AND DISCUSSION

### Influence of PtcCo(II) residue on the kinetics of formation and the stability of complex between the DNA target and the oligonucleotide conjugate

#### Stopped-flow kinetics

The influence of PtcCo(II) residue on the duplex formation between target **P** and oligonucleotide part of the conjugate was first investigated using the conjugate **X** and a nonmodified 10 nt oligonucleotide **N**. Formation of **PX** and **PN** was studied by stopped-flow kinetics with optical density detection at 255 nm.

**Figure 2:**
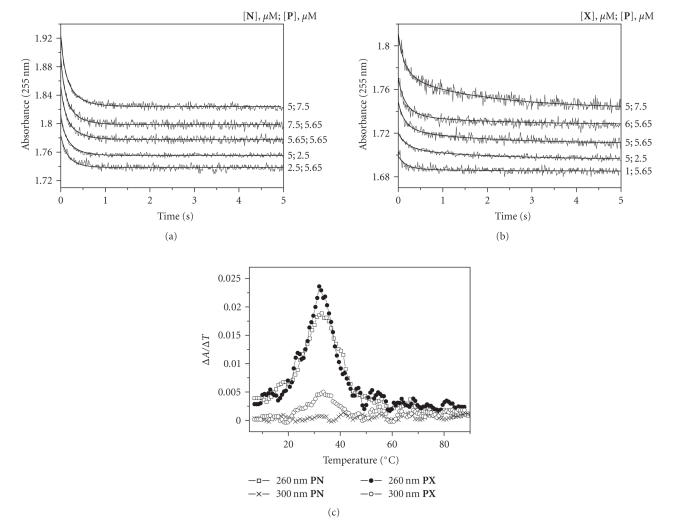
The kinetic curves of the formation of complexes between the target **P** and the oligonucleotide **N** (a) or the conjugate **X** (b); (c) the differential melting curves of the complexes **PN** and **PX**.

As can be seen from Figures 2(a) and 2(b), the kinetic curves for the interaction of **P** with **X** and **N** were different. In the case of **PN** formation, a sharp decrease in the absorption was observed between 0 and 1 s, followed by a plateau phase after 1 s. When **PX** was formed, a slow decrease in the optical density after 1 s was detected. It should be noted that **N** caused a more pronounced change in the optical density at 255 nm compared with **X**. For example, at [**P**] = 7.5×10^–6^ M (the concentrations of **X** and **N** were 5.0×10^–6^ M) the change of the absorbance was ∼0.1 in the case of **PN** and ∼0.05 with **PX**.

**Scheme 2:**
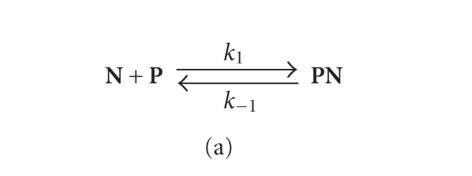

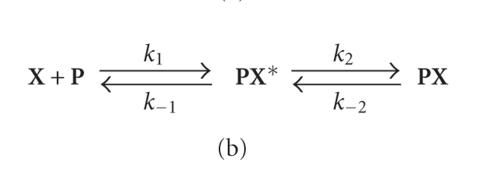
The formation of the complexes between the target **P** and the oligonucleotide **N** (a) or the conjugate **X** (b).

The kinetic curves of **PN** formation were fitted to Scheme 2(a) using DynaFit software. The theoretical curves correlated well with the experimental data. The calculated rate constants are presented in Table 1.

In the case of **PX**, Scheme 2(a) did not describe the slight decrease of the optical density after 1 s. These data were treated using Scheme 2(b) containing two equilibria. The calculated constants are presented in Table 1. Accordingly, the process of the complex formation between the conjugate and target oligonucleotide can be described by a two-stage mechanism. The overall association constant for the formation of **PX** was in a close agreement with the overall constant for the **PN** formation, indicating that the phthalocyanine residue did not influence the complex stability. It was shown earlier [18] that the association constant for the complex formed by a conjugate of a 8 nt oligonucleotide with Co(II)-phthalocyanine and a 12 nt oligonucleotide target is 15-fold higher than that for the complex formed with the nonmodified 8 nt oligonucleotide and the 12 nt target. However, the stabilization/destabilization effects may depend on the lengths and sequences of the oligonucleotides used. In our case when a 20 nt oligonucleotide was used as the target, no stabilization of its duplex with the conjugate was observed.

The process of DNA duplex formation includes two steps: nucleation and zipping of the duplex. The first stage is rate-limiting and proceeds with the rate constant about 1 × 10^6^ – 1 × 10^7^ (M × s)^–1^ [19]. In the case of the conjugate containing two binding parts, their influence on the complex formation could be rather complicated. Free porphyrins and their analogs are known to interact with DNA through either intercalation or outside binding [20, 21]. The binding mode depends both on the nucleotide sequence and substitutions in the porphyrin moiety. It was shown [18] that Co(II)Ptc interacts with DNA chain. Thus, both fragments of the oligonucleotide-phthalocyanine conjugate can interact with the target oligonucleotide. The following mechanism of **PX** formation is possible. As can be seen from Figure 2, the optical density decreased quickly until 1 s when both **X** and **N** were binding to **P**. This decrease is due to the hypochromic effect arising from the formation of Watson-Crick base pairs between the oligonucleotides. Thus, the slow second stage present in the case of **PX** is likely caused by the phthalocyanine residue. Table 1 shows that rate constants *k*_1_ for **PN** and PX* are similar but the values of *k*_–1_ differ twofold, suggesting that PX* is less stable than **PN**. In this case the stability of the complex depends on the number of the Watson-Crick base pairs formed at the first moment. The smaller change in the optical density during **PX** formation corroborates this conclusion because the change in the absorbance is proportional to the number of Watson-Crick base pairs. One can suggest that at the first stage only a few base pairs are formed between the conjugate and the oligonucleotide target and the phthalocyanine moiety of the conjugate sterically hinders base pairing. This effect may be due to the interaction of the phthalocyanine residue with 3-4 heterocyclic bases in the conjugate itself, or with bases in the target. In any case, incompletely “zipped” duplex PX* is likely to be formed at the first step. The slow phthalocyanine displacement at the second step leads to a formation of the fully complementary **PX** complex.

##### Melting curves

To determine the thermodynamic parameters of duplex stability, the UV melting curves were examined. Single-stranded oligonucleotide **P** yielded no visible transition in the differential melting curves recorded at 26 nm, indicating that **P** has no stable secondary structure. The same result was obtained when the conjugate was heated.

The differential melting curves of the complexes **PN** and **PX** had a single well-defined transition with T_m_ = 32.6 and 32.9°C, respectively (Figure 2(c)). Registration of melting curves at 300 nm permitted us to detect the interaction of phthalocyanine moiety with the target. The shape of the differential melting curve at 300 nm and the location of its maximum were identical to the shapes of the curves recorded at 260, 270, and 280 nm. Consequently, the melting curves at 260–280 nm and 300 nm described the same process, showing that the “melting” of the phthalocyanine residue occurred simultaneously with the melting of the oligonucleotide duplex.

The thermodynamic parameters of dissociation of **PN** and **PX** (ΔS^0^, ΔH^0^, ΔG^0^_298_) were determined using Simplex software. The obtained data are listed in Table 2. The association constants were close to those obtained from the stopped-flow experiments. Taken together, the results obtained by thermal denaturation method were in agreement with the data obtained by the fast kinetic method: the phthalocyanine residue does not influence the stability of the complex between the conjugate and the target.

### Degradation of the phthalocyanine residue in the presence of hydrogen peroxide

Treatment of the conjugate with hydrogen peroxide resulted in a destruction of the phthalocyanine residue. This process is accompanied by a decrease in the optical density at 650–750 nm and at 320 nm, corresponding to the Q-bands and the Soret band, respectively. We have studied the kinetics of the phthalocyanine destruction by recording the absorbance at 682 nm.

**Figure 3:**
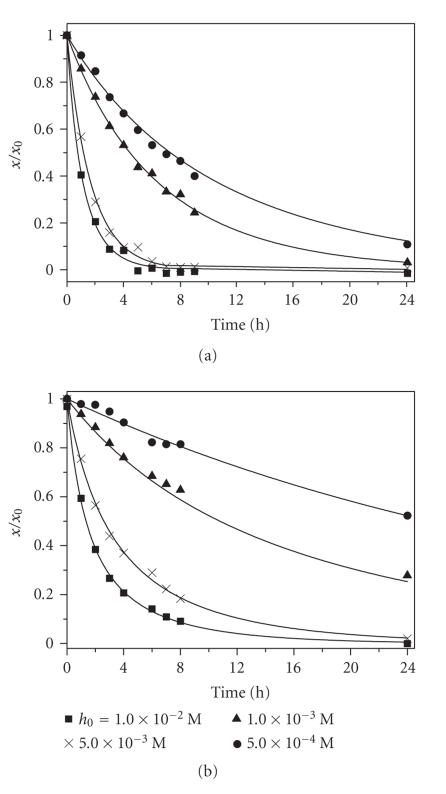
The time course of the PtcCo(II) residue degradation; (a) *x*_0_ = 0.8 × 10^–5^ M, (b) *x*_0_ = 1.0 × 10^–5^ M.

The kinetic curves corresponding to different concentrations of H_2_O_2_ are presented on Figure 3. Total degradation of the PtcCo(II) group was observed for high concentration of hydrogen peroxide (1.0 × 10^–2^ and 5.0 × 10^–3^ M). The reaction did not reach its plateau by 24 h at low concentrations of oxidizer (1.0×10^–3^ and 5.0×10^–4^ M). The repeated addition of H_2_O_2_ to the reaction mixture after 24 h led to a further decrease in the optical density.

The degradation of PtcCo(II) residue by H_2_O_2_ was accompanied with catalytic decomposition of hydrogen peroxide. The experimental data were satisfactorily described assuming that the destruction of conjugate followed second-order kinetics (first-order with respect to both components). The catalytic decomposition of H_2_O_2_ was described as the third-order reaction (first-order by the conjugate and second-order by hydrogen peroxide). The values of rate constants *k_d_* (conjugate destruction) and *k_h_* (catalytic decomposition of H_2_O_2_) obtained by fitting using Scientist software were *k_d_* = (2.2 ± 0.2) × 10^–2^ (M × s)^–1^ and *k_h_* = (2.5 ± 0.5) × 10^3^ (M^2^ × s)^–1^.

Kremer in his work has shown [22] that the catalytic decomposition of H_2_O_2_ by hemin is also second-order with respect to hydrogen peroxide. The process included formation of a primary heme-H_2_O_2_ complex following by coordination of the second H_2_O_2_ molecule and the catalytic act of hydrogen peroxide decomposition. It is quite possible that the catalysis of H_2_O_2_ decomposition by the phthalocyanine Co(II) proceeds similarly.

Comparing our results with those obtained for the modification of a target with a conjugate of a 8 nt oligonucleotide with a Fe(III)-protoporphyrin IX (hemin) group in the presence of H_2_O_2_ [23], we conclude that the rate constant of the phthalocyanine residue destruction was ∼120-fold lower than that of the hemin group. The destruction of the porphyrin system was the major cause of the low extent of target modification, which did not exceed 33%.

### Modification of the target oligonucleotide

**Figure 4:**
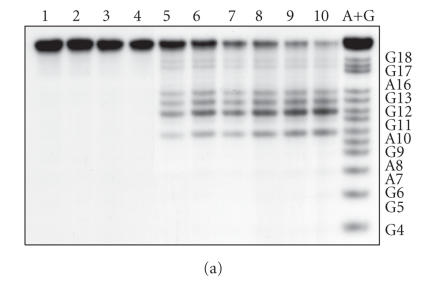

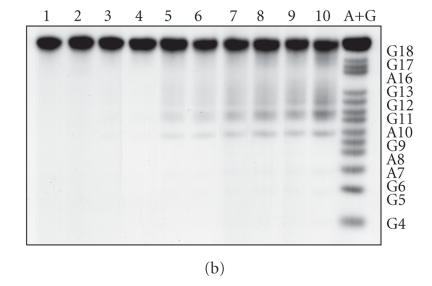

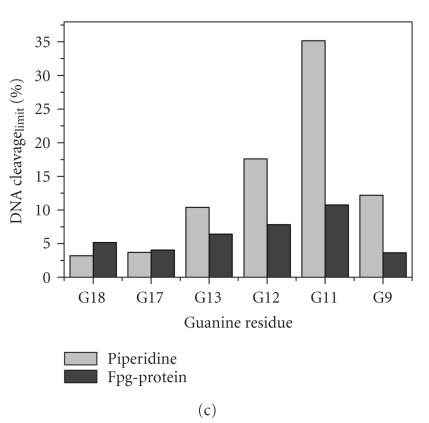
The autoradiograms of the denatured 20% PAAG of the [^32^P]-labeled target modified by the conjugate (1.0 × 10^–5^ M) in presence of H_2_O_2_ (1.0 × 10^–2^ M) after treatment with 1 M piperidine (a) or Fpg protein (b). The sample in lane 1 did not contain both the conjugate and H_2_O_2_. The samples in lanes 2 and 3 did not contain the conjugate or H_2_O_2_, respectively. Time points shown are 0, 1, 2, 3, 5, 8, and 24 hours (lanes 4–10). (c) The distribution of the base modifications in the target.

Kinetics of DNA modification within **PX** was studied by following the time course of cleavage of the [^32^P]-labeled target at different times. No direct cleavage of the target strand was observed. Alkali-labile modifications (abasic sites and oxidized deoxyribose) were revealed by piperidine treatment of the DNA target. To digest the alkali-resistant products oxidized at deoxyguanosine residues (eg, 8-oxoguanine), the samples were treated with Fpg protein. Typical autoradiograms are presented in Figures 4(a) and 4(b). The yields and positions of the modified bases in the target were determined (Figure 4(c)). The modification occurred preferentially at guanine residues in the region G^9^–G^13^, indicating that the guanine bases close to the source of ^•^OH radicals are the most susceptible, and that the stretch of **P** forming the duplex with the oligonucleotide part of the conjugate is protected from ^•^OH radicals by **X**. These observations suggest that the preferential modification of **P** at G^9^–G^13^ is due to the attack by ^•^OH radicals before their diffusion in solution. Similarly localized damage was observed for irradiated DNA-Cu^2+^ molecules [24].

**Figure 5:**
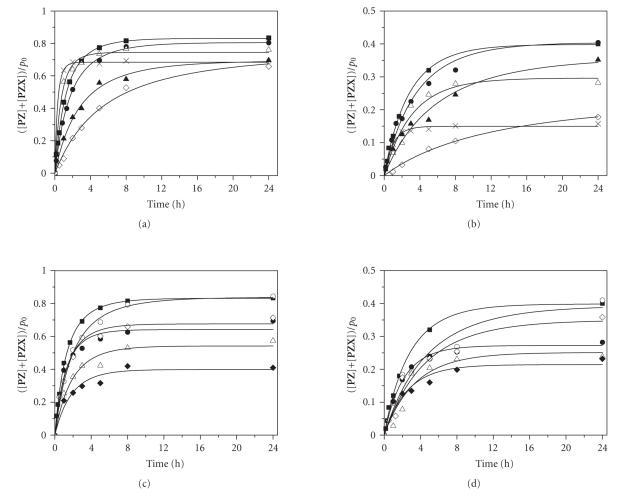
The kinetic and theoretical curves of the modification process of the target. (a) and (b) *x*_0_ = 5.6 × 10^–6^ M, *h*_0_ = 1.0 × 10^–1^ M(×), 2.5 × 10^–2^ M(Δ), 1.0 × 10^–2^ M (■), 5.0 × 10^–3^ M (•), 2.0 × 10^–3^ M (▴), 1.0 × 10^–3^ M (◊). (c) and (d) *h*_0_ = 1.0 × 10^–2^ M, *x*_0_ = 1.0 × 10^–5^ M (∘), 5.6 × 10^–6^ M (■), 2.8 × 10^–6^ M (◊), 1.5 × 10^–6^ M (•), 0.7 × 10^–6^ M (Δ), 0.4 × 10^–6^ M (⧫). The modifications were revealed by treatment with 1 M piperidine (a) and (c) or Fpg-protein (b) and (d).

The total modification extent of 80% was achieved with piperidine treatment and 40% with Fpg treatment. Since some of the modification products revealed by Fpg could be also determined by piperidine, the total modification extent was at least 80% and likely between 80 and 100%. The time courses of modification are shown in Figure 5.

**Scheme 3:**
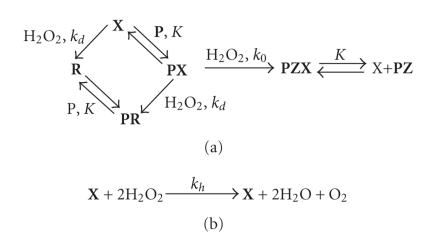
The oxidation modification of the target with conjugate in the presence of H_2_O_2_. (In this scheme, **P** is the target oligonucleotide, **X** is the conjugate, **R** is the oxidation product of the phthalocyanine moiety possessing the same affinity to the target as the conjugate, **PZ** is the modification product, **PX**, **PR**, and **PZX** are respective complexes, and *K*, *k_d_*, *k*_0_, and *k_h_* are respective association and rate constants.)

To describe the oxidative modification of the target by the conjugate in the presence of hydrogen peroxide, Scheme 3 was proposed. The experiments were carried out under the conditions where *x*_0_ ≫ *p*_0_. The large excess of the conjugate over the target was used in order to obtain complete binding of **P** into **PX** and to achieve the maximal level of target modification. In addition, as **PX** ≪ **X**, the decomposition of H_2_O_2_ by **PX** was disregarded.

The kinetic curves were satisfactorily described with Scheme 3. The values of rate constants *k_d_* = 2.2 × 10^–2^ (M × s)^–1^, *k_h_* = 2.5 × 10^3^ (M^2^ × s)^–1^, and *K* = 3.0 × 10^–6^ M^–1^ determined previously (see Tables 1 and 3) were taken for fitting procedure using Scientist software. The rate constants of the target modification (*k*_0_^Fpg^ and *k*_0_^Pip^) were fitted in this case. The values of *k*_0_ were found to be dependent on the type of product analysis (Table 3). This means that differentproducts of guanine oxidation identified by piperidine and Fpg treatments accumulated with different rates. The piperidine treatment gave a higher total modification extent and *k*_0_ value than the Fpg treatment.

**Figure 6:**
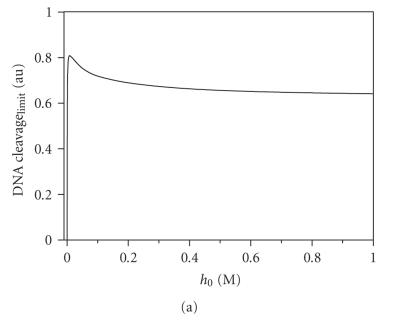

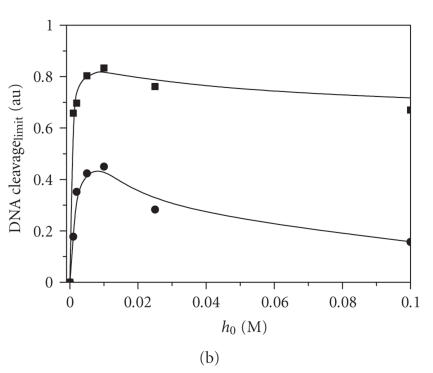

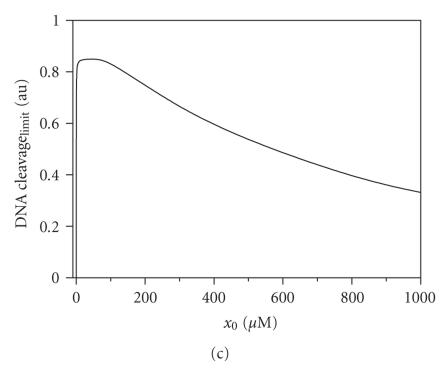

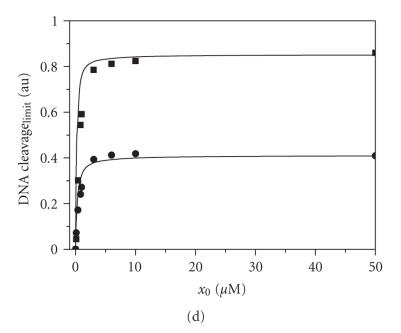
The dependence of the modification extend of target by the conjugate. (a) and (c) The theoretical curves were obtained by simulation of the kinetic process of the DNA modification using Scheme 3; (b) and (d) represent the experimental observed curves of the dependence of the modification product on *h*_0_ or *x*_0_, respectively. The modifications were revealed by treatment with 1 M piperidine (■) or Fpg protein (•).

When the DNA target is oxidized in the presence of a catalytically active oligonucleotide derivative and H_2_O_2_, several competing processes take place. First of all, the target is modified within the duplex with conjugate. Second, the catalytic group is damaged in the side reaction with hydrogen peroxide. Third, H_2_O_2_ is catalytically decomposed into O_2_ and H_2_O. The simulation according to Scheme 3 has shown that the dependence of the product modification on the initial concentrations of H_2_O_2_ should have a maximum (Figure 6(a)), and its existence was experimentally confirmed (Figure 6(b)).

The simulation of the dependence of the modification depth on the initial concentration of the conjugate also resulted in the curve with a maximum at about 30–100 *μ*M (Figure 6(c)). The decrease in the modification extent with the increase in the conjugate concentration was possibly connected to the catalytic H_2_O_2_ decomposition being the predominant process at these concentrations of the catalyst. Experimentally we could not achieve such high concentrations of conjugate; when they were varied from 0 to 50 *μ*M; a predicted hyperbolic curve was observed (Figure 6(d)).

Our results show that the PtcCo(II) group in the oligonucleotide conjugate is able to modify target DNA in the presence of H_2_O_2_ as an oxidant. Since this group acts as a catalyst of oxidation, such conjugates may be considered as artificial enzymes, the synthetic analogs of peroxidases. Hydrogen peroxide can be formed inside the cell in some endogenous processes (respiratory burst in mitochondria, oxidative stress, inflammation, etc, [25, 26]) and can be involved in oxidation of target nucleic acids.

Oxidative modification of DNA with the PtcCo(II)-oli-gonucleotide conjugate is accompanied by destruction of the phthalocyanine macrocycle by the oxidant and catalytic decomposition of the oxidant. The bell-shaped dependence of the modification efficiency on the H_2_O_2_ concentration suggests that these three processes are in competition. In comparison with the previously studied Fe(II)-porphyrin-oligonucleotide conjugate [23], the phthalocyanine group is more stable to degradation. This property is useful for the design of oligonucleotides-based drugs, which can be promising candidates for cancer therapy.

## Figures and Tables

**Table 1:** Rate and association constants for formation of complexes **PN** and **PX**.

Complex	*k*_1_ ((*μ*M × s)^–1^)	*k*_−1_ (s^−1^)	*k*_2_ (s^−1^)	*k*_–2_ (s^−1^)	* *K* ((*μ*M)^−1^)

**PN**	(9.6 ± 0.4) × 10^−1^	0.4 ± 0.1	—	—	2.6 ± 0.4
**PX**	(7.6 ± 0.2) × 10^−1^	0.8 ± 0.1	(4.0 ± 0.3) × 10^−1^	(2.3 ± 0.2) × 10^–1^	3.0 ± 0.4

* *K* = ∑^*i*=2^_*i*=1_ Π^*j=i*^_*j*=1_ *K_j_*.

**Table 2:** The thermodynamic parameters and association constants for formation of complexes **PN** and **PX**.

Complex	T_m_ (°C)	–ΔS^0^ (cal/(mol × K))	–ΔH^0^ (kcal/mol)	–ΔG^0^_298_ (kcal/mol)	* *K* ((*μ*M)^–1^)

**PN**	32.6 ± 0.2	168.8 ± 6.8	59.5 ± 2.0	9.2 ± 0.1	5.5 ± 1.1
**PX**	32.9 ± 0.2	204.2 ± 12.8	70.0 ± 3.9	9.1 ± 0.1	4.7 ± 0.9

*K = exp(–ΔG^0^_298_/RT).

**Table 3:** The rate and association constants obtained from modification data.

	Piperidine treatment	Fpg protein treatment

*K* ((*μ*M)^–1^)	(3.0 ± 0.4)	
*k_d_* ((M × s)^–1^)	(2.2 ± 0.2) × 10^–2^	
*k_h_* ((M^2^ × s)^–1^)	(2.5 ± 0.5) × 10^3^	
*k_0_^i^* ((M × s)^–1^)	(4.2 ± 0.6) × 10^–2^	(1.2 ± 0.2) × 10^–2^
